# Technology of percutaneous cannulated screw implantation using screw view model of navigation in Garden type I of femoral neck fracture: A case report

**DOI:** 10.1097/MD.0000000000015591

**Published:** 2019-05-24

**Authors:** Tong Yu, Bao-Ming Yuan, Xi-Wen Zhang, Dong-Sheng Wang, Yi-Kun Jiang, Rong-Peng Dong, Ming-Yang Kang, Xin Zhao, Jian-Wu Zhao

**Affiliations:** a Department of Orthopaedics; b Department of Gynecology, The Second Hospital of Jilin University, Changchun, Jilin Province, China.

**Keywords:** cannulated screw, femoral neck fracture, navigation, percutaneous

## Abstract

**Rationale:**

The aim of the present study was to assess the efficacy and safety of percutaneous cannulated screw (PCS) implantation assisted by screw view model of navigation (SVMN) to treat femoral neck fracture (FNF).

**Patient concerns:**

A 42-year-old male patient suffered from a high falling injury, causing pain, swelling, deformity, and limited mobility on his right hip.

**Diagnoses:**

He was diagnosed with Garden type I of FNF.

**Interventions:**

PCS implantation assisted by SVMN was used to treat fracture of femoral neck in this patient.

**Outcomes:**

The follow up lasted for 48 months. A total of 3 screws were inserted into femoral neck, all exhibiting excellent position. The mean screw deviation was 0.43° and 5.73° of femoral neck-shaft and anteversion angle, respectively. The guide wire drilling attempt of each screw was one-time. The fluoroscopic time lasted 6.3 minutes, the Harris hip scores improved from 67 to 88, and the blood loss was 35 mL. It took 11.7 minutes for designing the screws, 13.9 minutes for implanting the guide wires, and 37.3 minutes for placing the screws. No clinical complications were found during 48-month follow-up visit, including head penetration, implant failure, fracture nonunion, and femoral head osteonecrosis.

**Lessons:**

The study revealed that SVMN is conducive to the PCS insertion for FNF. Our lesson is that the FNF must be well reduction before SVMN assisted PCS placement.

## 1 Introduction

With the aging of the global population, hip fractures will lead to an increase in emergency admission and surgery.^[1,2]^ Urgent surgical treatment is often required to avoid femoral head osteonecrosis and fracture nonunion.^[3]^ Percutaneous cannulated screw (PCS) fixation is considered an acceptable method for treating femoral head fractures because of its own advantages, including minimally invasive, short operation time, and good stability. Accurate cannulate screw placement is considered the most vital manipulation to avoid surgical complications.^[4]^

Cannulate screw placement by freehand for treating femoral neck fracture (FNF) obtained outcomes that were considered satisfactory. However, the complication rate of freehand cannulate screw placement reaches 12.5% to 55%.^[4,5]^ Measures to facilitate PCS implantation include computer-assisted surgery,^[6,7]^ robot-navigated surgery,^[8]^ and guide wire aiming device surgery.^[9]^ Yet, they are accompanied with many complications, for example, femoral head osteonecrosis,^[10]^ fracture nonunion,^[3,11–13]^ head penetration,^[4]^ and implant failure.^[14]^

To reduce the incidence of clinical complications, we applied a screw view model of navigation (SVMN) technology in the operation of non-displaced FNF. In this screw view model, the navigation monitor displays the static axial, sagittal, and coronal three dimensional computed tomography (3D CT) of FNF with designed screws, which makes the manipulation simple and accurate. To the best of our knowledge, the application of this novel technology with PCS placement has been rarely reported.

## 2 Ethical approval

The Second Hospital of Jilin University, Changchun, China, approved the study and the institutional guidelines for the care and treatment of patients were rigorously followed. Informed written consent was obtained from the patient for publication of this case report and accompanying images. (2019) Research and Inspection No. (007).

## 3 Case report

### 3.1 Patient characteristics

A 42-year-old male patient suffered a high falling injury (Table 1), causing pain, swelling, deformity, and limited mobility on his right hip. Physical examination revealed that the right hip was slightly swollen, there existed slight tapping pain around the greater trochanter, the midpoint tenderness of the inguinal ligament was positive, and the flexion of the hip was obviously limited. No significant bone rub was touched. Bilateral lower limbs had normal sensation, the temperature of lower extremity skin was normal, the pulsation of dorsal pedal artery can be touched, and the muscular tension of both lower extremities was normal.

**Table 1:**
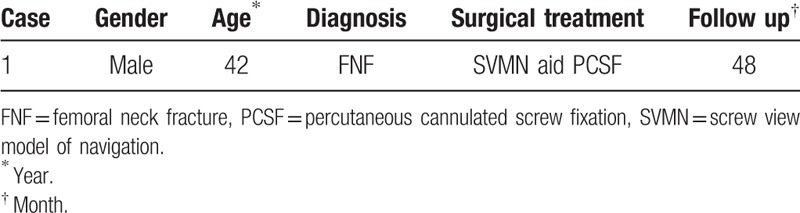
Basic characteristics of the patient.

**Figure 1:**
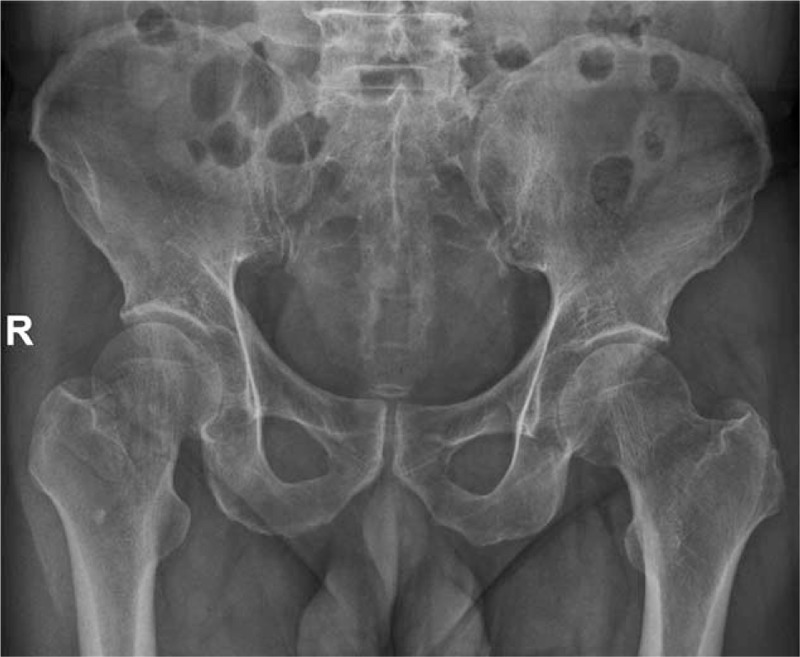
X-ray of right hip. A 42-year-old male patient suffered from a high falling injury. According to the preoperative x-ray image, the discontinuity of bone in the right femoral neck was observed, and femoral neck fracture was initially diagnosed.

**Figure 2:**
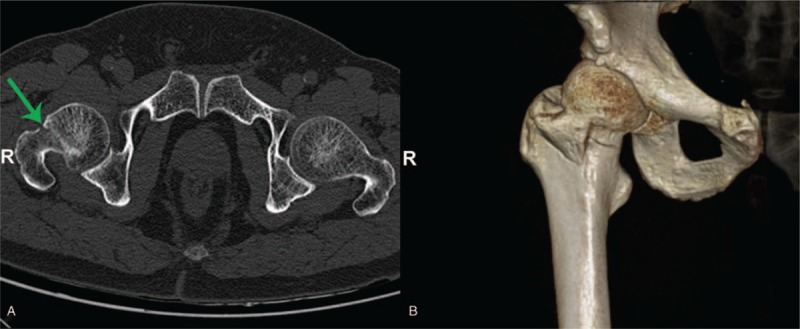
Preoperative CT of the right hip was obtained. (A) Axial plane image of right hip showed that the right femoral neck bone was discontinuous and linear translucent (green arrow), (B) three dimensional CT image of the injured right hip. CT = computed tomography.

The hip x-ray exhibited the discontinuity of bone in the right femoral neck (Fig. 1). Three-dimensional (3D) CT images showed that the right femoral neck bone was discontinuous and linear translucent (Fig. 2). The patient was primarily diagnosed as FNF. In accordance with Garden typing,^[15]^ it was classified as Garden I.

**Figure 3:**
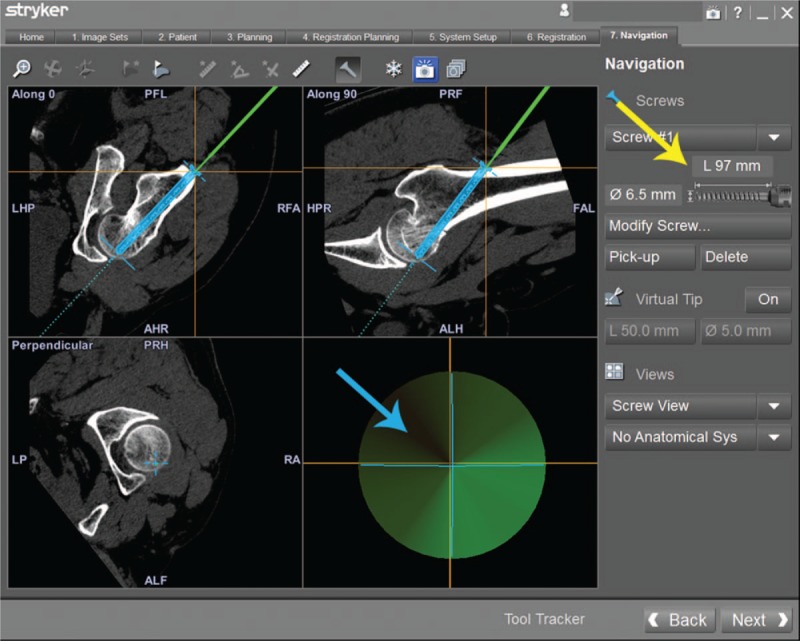
Screw designing and insertion. The length, diameter (yellow arrow), and the best trajectory of screws were determined after image acquisition. Intraoperative the SVMN was selected at workstation. When the right lower corner of the image shows green (blue arrow), and it would the best time to implant a guide needle. SVMN = screw view model of navigation.

The whole hip was scanned by CT preoperatively, and the result was shown in Fig. 2. The image information was recorded in the compact disc read-only memory, which could be read by a computer navigation workstation. The length, diameter, and the optimal trajectory of the PCS were designed at the navigation workstation preoperatively (Fig. 3).

### 3.2 Surgical procedures

The operation was performed under general anesthesia (intubation: propofol, 200 μg/kg, Qingyuan Jiabo Pharmaceutical Co., Ltd. China; fentanyl, 250 μg, RenFu LLC, YiChang, China; midazolam, 2 mg; maintenance: propofol, 0.2–0.5 mg/kg/h, Enhua Pharmaceutical Limited by Share Ltd., JiangSu, China). Short-acting muscle relaxants were provided only during the intubation. When the anesthesia worked, the patient was placed in the supine position.

First, a patient tracker (Stryker Leibinger GmbH & Co., Freiburg, Germany), operated with the Navigation System II-CART II with SpineMap 3D 2.0 software (Stryker Navigation, Kalamazoo, MI), was outfitted on the iliac crest at the beginning of the operation. The system's C-arm tracker, patient tracker, and guide needle sleeve tracker were all activated. After 190° scanning was performed at the center of the femoral neck, 3D images of the lesion were captured. Subsequently, preoperative and intraoperative CT images were matched to provide clear guidance for the guide needle sleeve.

Second, the screw view mode was selected in the navigation workstation. Besides, the position of the guide needle sleeve device was adjusted until the direction of guide needle sleeve completely complied with the planning PCS trajectory, and the guide wire was placed once the right lower corner image showed green (Fig. 3). At the end of the operation, cannulate screws were inserted sequentially through the implanted guide wire.

**Figure 4:**
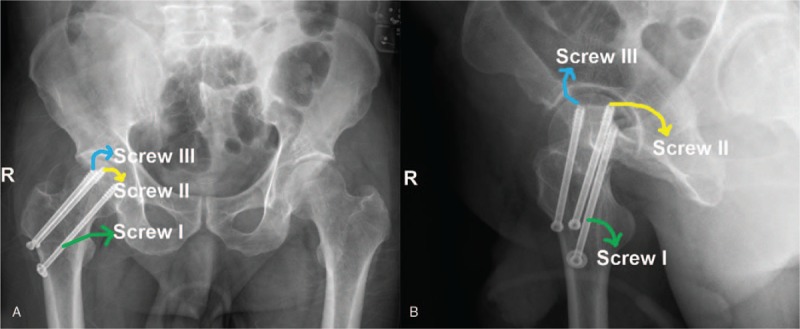
Postoperative x-ray of hip was examined. (A) Anteroposterior hip radiograph and (B) lateral hip radiograph.

**Figure 5:**
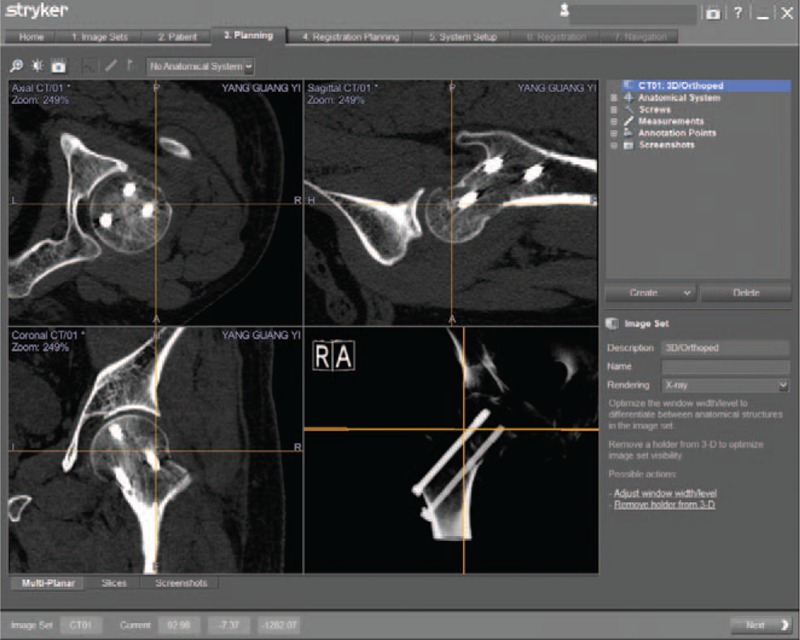
Postoperative CT of the right hip was scanned. Image data input into computer navigation showed that all the screws were in good position without penetrating the cortex. CT = computed tomography.

Finally, x-ray and CT scan were performed to verify whether the screw was in good position (Figs. 4 and 5). Postoperatively, the right lower extremity was abducted 15° to 30° under the fixed anti-rotation shoes. Four weeks after surgery, patients could get out of bed with double crutches. Four to 6 months after the operation, the patient could walk without crutches after fracture healing.

### 3.3 Outcomes and follow-up

#### 3.3.1 Evaluation parameters

**Figure 6:**
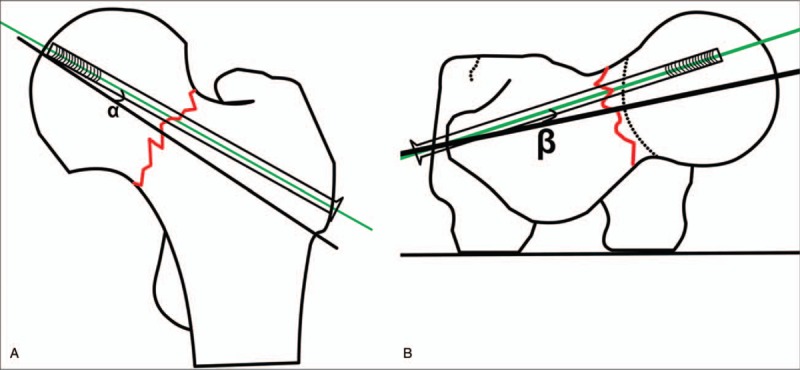
The deviation of screw's femoral neck-shaft angle and anteversion angle were calculated. (A) ∠*α* represents the deviation of femoral neck-shaft angle, (B) ∠*β* represents the deviation of anteversion angle.

**Figure 7:**
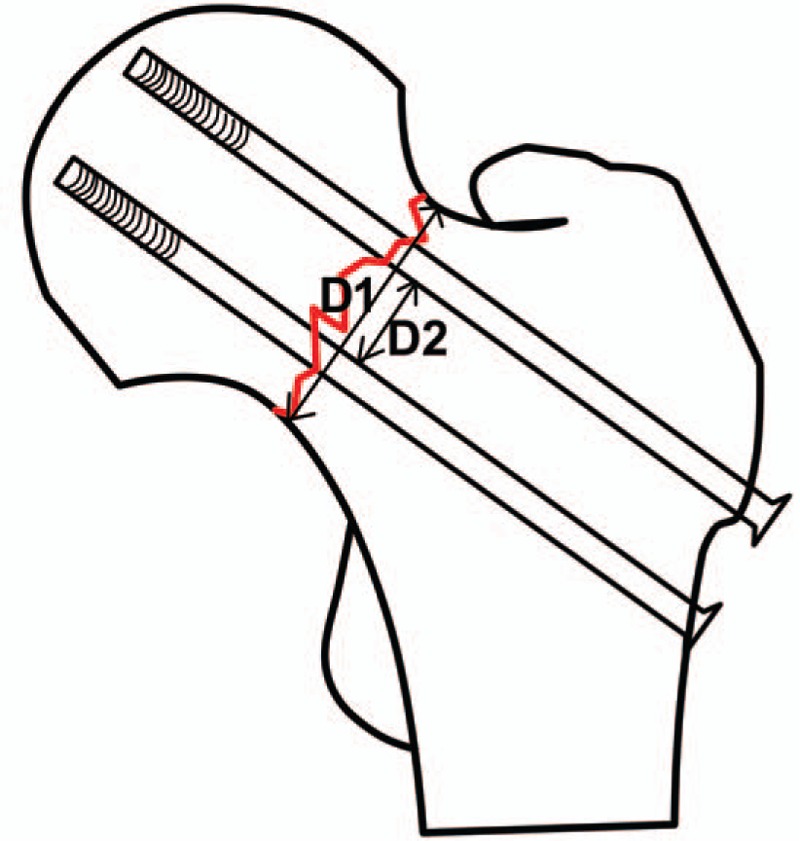
Neck coverage area of the femoral neck was calculated. Neck coverage area = D2/D1.

Position of cannulate screw in femoral neck: grade 0 was defined as excellent (the distance from the outer edge of the screw to the cortex of the femoral neck was 2–5 mm), grade 1 as good (>5 mm), grade 2 as general (<2 mm), and grade 3 as bad (penetration the cortex of femoral neck)^[16]^ (Table 2). The deviation of each screw's femoral neck-shaft angle and anteversion angle: the angles between the longitudinal axis of the 3 cannulate screws and the axis of the femoral neck were measured from the anteroposterior and lateral radiographs, respectively (Fig. 6, Table 3). Neck coverage area of the femoral neck was calculated^[4]^: neck coverage area = D2/D1. D1: the diameter of the femoral neck at the fracture level. D2: the distance from the inferior border of the most distal screw to the superior border of the proximal screw (Fig. 7, Table 3). Furthermore, the time for designing the screws, implanting the guide wire and inserting the screws, the amount of bleeding, the guide wire drilling attempts, fluoroscopic time and Harris hip scores were assessed (Table 2).

**Table 2:**
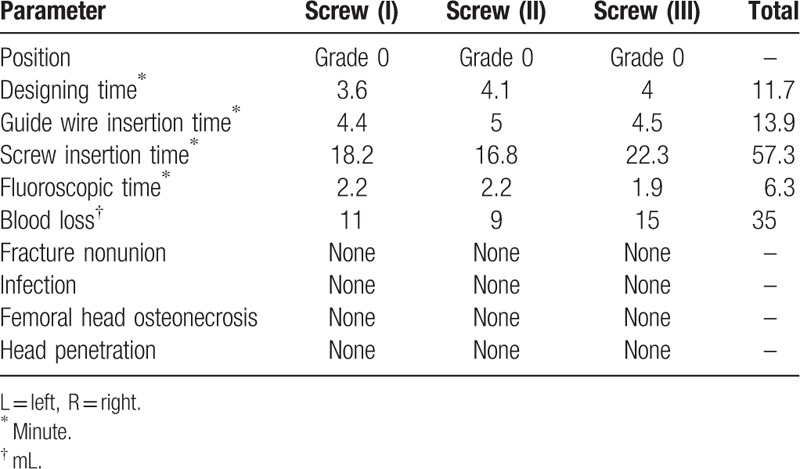
Basic characteristics of cannulated screws.

**Table 3:**
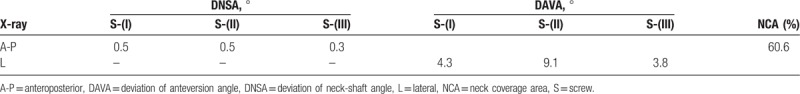
Evaluation parameters of cannulated screw position.

## 4 Results

The postoperative 3D CT shows that the 3 implanted screws were all in position of grade 0. The follow up visit lasted for 48 months. The mean angle of screw deviation was 0.43° and 5.73°, respectively, in the femoral neck-shaft angle, anteversion angle, and details are listed in Table 2. The mean coverages of femoral neck measured on anteroposterior and lateral radiographs were 90% and 85%, respectively (Table 2).

It took 11.7 minutes for designing the screws, 13.9 minutes for implanting the guide wires, and 37.3 minutes for placing the screws. The frequency of drilling attempts of each screw was one-time, intraoperative blood loss was 35 mL, the fluoroscopic time lasted 6.3 minutes and Harris hip scores improved from 67 to 88. No clinical complications, such as fracture nonunion, femoral head osteonecrosis, head penetration, and implant failure, were found during 48-month follow-up (Table 2).

## 5 Discussion

Perfect cannulate screw placement should satisfy the following requirements.^[4]^ First, complete an entry point for the 3 screws above the distal end of the lesser trochanter. Second, avoid femoral head penetration. Third, achieve the maximal spread of the 3 parallel screws in the femoral neck. However, it is hard to insert the guide needle at the perfect position. Accordingly, complications (e.g., multiple drills, increased fluoroscopy time, uneven screw distribution, head perforation, and failure of internal fixation) may occur during the operation.^[3–5,10,12–14]^ Accordingly, appropriate placement of guide needles is vital to treat FNF with PCS.^[17]^ The use of a SVMN technique was explored to assist in a PCS insertion.

The plan function of computer navigation system help the surgeon calculate the length, angle, entry point, and plan trajectory of the screw preoperatively, and SVMN exhibits a perfect screw position on the static axial, sagittal, and coronal image of 3D CT.^[18,19]^ The navigation of the guide needle's direction in the femoral neck can be clearly seen through the image intraoperatively. As a result, the 3 cannulate screws achieved better parallelism and spread with fewer failed attempts. In the present study, the position of the 3 screws were all classified as grade 0 exhibiting small deviation angle, good spread, and parallelism. Each screw was successfully inserted at its first attempt. Compared with the results of previous findings,^[7]^ the drill attempts here were less, and the fluoroscopy time was shorter. No surgical complication was shown here on follow-up, such as no head penetration and implant failure. This positive result was attributed to the use of the navigation system for preoperative planning and the guidance of PCS insertion with a SVMN.

Fracture nonunion and femoral head osteonecrosis are common complications after FNF surgery with PCS. Previous literatures showed that the incidence of fracture nonunion was ranged from 10% to 47%,^[3,20–22]^ and femoral head osteonecrosis was up to 17% to 45% with PCS.^[3,20,22,23]^ The medial femoral circumflex artery and lateral femoral circumflex artery provide 90% of the blood supply to the femoral head.^[24]^ Fractures of the femoral neck can cause arterial damage, thereby leading to avascular necrosis of the femoral head. Rahman et al^[25]^ reported that the risk of avascular necrosis was associated with the degree of displacement. In the present study, patients with Garden type I of FNF found no nonunion and femoral head osteonecrosis during follow-up. The reasons for this good result were concluded. On the one hand, the medial and lateral femoral circumflex arteries were not seriously damaged since the displacement of the fracture is small. On the other hand, this was because accurate screw placement avoids iatrogenic vascular injury and stable internal fixation.

Our SVMN technology has the following advantages in design: first, it avoids the effects of multiple drilling on blood supply of femoral head. Second, intraoperative fluoroscopy time was reduced, and radiation exposure of patients and medical staff was reduced. Third, accurate placement of cannulated screw (excellent position score, small deviation of neck-shaft angle and anteversion angle, good distribution rate) cannot only increase the stability of fracture fixation, but also avoid screw cutting bone cortex.

For the drawbacks of this technology, it has narrower range of indications in the navigation system, it is only suitable for minimal dislocation of FNF, for example, Garden type I and Garden type II, and Garden Type III and Garden IV of FNF require good reduction before SVMN guided PCS fixation. Furthermore, the sample size here is very small, and more samples are required to assess the safety and effectiveness of this technology.

In conclusion, the PCS insertion assisted by SVMN technology is a simple and highly feasible procedure. Our lesson is that the FNF must be well reduction before SVMN assisted PCS placement.

## Acknowledgments

The authors gratefully acknowledge the cooperation of the doctors and nurses in the operating room.

## Author contributions

**Conceptualization:** Bao-Ming Yuan, Dong-Sheng Wang.

**Data curation:** Yi-Kun Jiang.

**Investigation:** Rong-Peng Dong.

**Methodology:** Xin Zhao.

**Project administration:** Dong-Sheng Wang, Ming-Yang Kang.

**Supervision:** Jianwu Zhao.

**Validation:** Yi-Kun Jiang.

**Writing – original draft:** Tong Yu, Bao-Ming Yuan, Xi-Wen Zhang, Rong-Peng Dong, Xin Zhao, Jianwu Zhao.

**Writing – review & editing:** Tong Yu.
